# Medical Association of Central New York

**Published:** 1906-05

**Authors:** C. A. Greenleaf

**Affiliations:** Secretary of Rochester


### SOCIETY PROCEEDINGS.

### Medical Association of Central New York

Reported by Dr. C. A. GREENLEAF, Secretary, of Rochester.

THIRTY-EIGHTH Annual Session of the Medical Association of Central New York, held October 24, 1905, at the Lecture Room of the Buffalo Historical Society Building, Buffalo, N. Y.

DR. C. G. STOCKTON, President, in the Chair.

The president: The Buffalo Historical Society has very kindly extended to us the use of its commodious apartments in this building, which is almost historic in itself, and the President of the Society is here. I take pleasure in presenting MR. ANDREW LANGDON.

MR. LANGDON: I assure you that I had no thought your president would call upon me to say anything. Indeed it is to me very embarassing to appear before a body of scientists, professional gentlemen, in this way, but in the interest of the Historical Society I take pleasure in bidding you welcome. I also want you to feel that in coming here you are doing the Historical Society a very great honor, and certainly imparting a very great pleasure, and so I again say, I bid you a hearty welcome in the name of the Society. As they say in Mexico when they feel good-natured, "It is all yours." I hope you will feel entirely at liberty to go about the building and look at everything you like and ask any questions you feel inclined to of the attendants, who will show you every courtesy in their power. If by chance one of you should break a leg or arm we have all the modern appliances in the way of surgical implements in the case by the door and those are at your service.

The president: The Association appreciates very deeply the kindness shown us by the Buffalo Historical Society and the courtesy evinced by its president.

The following committees have been appointed, and in order that they may begin their duties I will announce them officially:

*Arrangements*: Chairman, Wm. C. Krauss, Buffalo; A. A. Hubbell, Buffalo; F. S. Crego, Buffalo; Lucien Howe, Buffalo; Chas. G. Stockton, Buffalo. *Business*: Chairman. Frank H. Stephenson, Syracuse; John Zimmer, Rochester; A. L. Benedict, Buffalo. *Credentials*: Chairman, Wm. A. Howe, Phelps, N. Y.; C. E. Congdon, Buffalo; W. W. Skinner, Geneva. *Ethics*: Chairman, Wm. B. Jones, Rochester; J. Henry Dowd, Buffalo; Wm. D. Johnson, Batavia. *Publication*: Chairman, Wm. C. Krauss, Buffalo; C. A. Greenleaf, Rochester; Wm. M. Brown,

Rochester. *Reception*: Chairman, C. C. Frederick, Buffalo; G. W. Wende, Buffalo; James W. Putnam, Buffalo; Allen A Jones, Buffalo; DeLancy Rochester, Buffalo; George F. Cott, Buffalo; Wm. C. Phelps, Buffalo; F. W. Hinkel, Buffalo; E. J. Meyer, Buffalo; G. W. York, Buffalo; J. Parmenter, Buffalo; Geo. L. Brown, Buffalo; C. Cary, Buffalo; M. D. Mann, Buffalo; A. W. Hurd, Buffalo; Roswell Park, Buffalo; H. C. Buswell, Buffalo; C. A. Wall, Buffalo; F. E. Fronczak, Buffalo.

The president called for the report of the treasurer.

Wm. M. Brown, Treasurer, presented his report, which is as follows:

| | | |
|---|---|---|
| 1904 | | |
| Oct. 18—Cash on hand | $271.32 | |
| Oct. 18—Dues collected | 234.00 | |
| Total | $505.32 | |
| DISBURSEMENTS | | |
| Oct. 18—Masonic Temple | | $10.00 |
| Oct. 18—C. A. Greenleaf, salary | | 10.00 |
| Oct. 18—Messenger boy | | 1.00 |
| Oct. 22—Interstate Printing Co. | | 3.75 |
| Oct. 22—Gillies Lithographing Co. | | 60.50 |
| Oct. 25—F. W. Parkhurst | | 37.00 |
| 1905 | | |
| Aug. 15—A. T. Brown Printing House | | 46.80 |
| Oct. 23—Postage and supplies | | 16.88 |
| | | $185.93 |
| Oct. 24—Balance on hand | $319.39 | |

It was moved and seconded that the report of the treasurer be accepted and placed on file.

Carried unanimously.

The president: Are there any other committees prepared to report?

Dr. Greenleaf, Rochester: The report of the committee on the revision of the constitution comes up for final vote; that is, the revision is to be acted upon today. It was held open last year, and in conformity to the old constitution has to lay over one year. The changes recommended are as follows:

Article 2, to read: The association shall meet annually on the third Tuesday of October, the meetings to be held in Rochester, Buffalo, and Syracuse in rotation, and in any other place by special invitation.

Article 3: To have Allegany, Cattaraugus, Chautauqua, Chemung, and Niagara added.

Article 8, to read: It shall be the duty of the secretary to keep an accurate record of the proceedings of the meeting of the association, to report all communications, to keep a list of the names of all members and delegates, and to send notices to members at least ten days previous to each meeting.

Article 10, to read: To "take charge of" in place of "procure."

Article 12, to read: "That may be required," in place of "it may see fit."

Article 15: To be stricken out as superfluous.

Article 16, to read: This association shall have power by vote at its regular meetings to expel any member for unprofessional conduct, or for acts derogatory to the honor and good name of the profession.

Article 19, to read: Members of any county society may have papers read for them by delegates on whatever subject they may choose.

Article 20, to read: Any member whose dues remain unpaid for a period of four years, may be dropped from the roll after due notification of such delinquency by the treasurer.

The president: The report of the committee on revision of by-laws has been read in general. Now it comes up for action at this meeting.

Dr. Greeleaf: These were passed upon as a whole last year. I move that these changes in the Constitution be adopted as a whole and the Secretary be empowered to make the change accordingly.

The president: The chairman of the committee on revision of by-laws moves that the by-laws, having been acted upon last year, and coming up this year for final action, shall be adopted and the committee be instructed to complete the by-laws by adding these amendments thereto. Are you ready for the vote?

Motion seconded and carried.

The president: Are there any other committees to report? If not, we will turn to the general program. Before reaching the next order I have a letter which I desire to read:

Oct. 21, 1905.

Dr. Chas. G. Stockton, President,
Buffalo, N. Y.

My Dear Doctor:—

At the last annual meeting of the Medical Association of Central New York, resolutions of sympathy in my misfortune were adopted. The kind and encouraging words of the members of the association, embodied in the resolutions, were brought to my bedside in the hospital. How much this expression of brotherly feeling meant to me at that trying time, can scarcely be put into words and I can only ask you to extend my sincere thanks and appreciation of the action taken.

I plan to be with you at the Buffalo meeting and enjoy the bountiful intellectual feast you have prepared for us. With kind regards and best wishes to all.

Sincerely Yours,
LOUIS A. WEIGEL.

The president: The regular order of business then, the literary part of the meeting, comes next, and I believe the president's address is the first thing on the program.

ALLEN A. JONES, of Buffalo: If I may be permitted before the president reads his address, I move a vote of welcome to members here from out of town who are not delegates of the Association, and all other members of the profession, that they be invited to take part in the discussions and all the exercises of the day.

Motion seconded and carried.

The president then read his address.

*(Published in the February No. of this Journal)*.

DAVID M. TOTMAN, of Syracuse, read a paper entitled "The Lower Extremity of the Ulna as a Factor in Colles' Fracture."

DISCUSSION.

L. A. WEIGEL, of Rochester: If there is any one thing more apparent than another in the study of cases of Colles' fracture it is the great variation in the character and extent of the injury suffered by the different patients. I have here a series of photographs, *x*-ray photographs of patients suffering from Colles' fracture. I think you will find that in about eighty per cent. there is an injury of the styloid, which is usually a complete separation, and not merely a dislocation, as has been described by Dr. Moore. We always recognise the value of Dr. Moore's work in this line, but I do not know whether before the days of the *x*-ray it was possible to demonstrate how this little process is turned up. I cannot agree with the speaker that every case of Colles' fracture should be, or could be, treated by the mode to which he has referred. We have only to look at this series of pictures, which I will pass around, to see the great variation in the line of fractures, as well as the distribution of fragments. I want to call attention also to the danger of unnecessary manipulation in certain forms of these injuries. You will find several in which there is no dislocation, but in which there is absolutely and undoubtedly a fracture present. By attempting to manipulate you will be very apt to distribute the fragments. You will never get them back in the position in which they were before, and the result will be disappointing in many cases. Dr. Totman has also called attention to another point, and that is the continued disability, or deformity, occurring after, sometime in these injuries. If I should proceed to make some remarks on that point I would simply be anticipating my own paper, and with Dr. Totman's permission, I would like to use his own photographs to demonstrate a condition which is not generally recognised.

DR. BENNINGHOFF, Bradford, Pa.: Mr. President, like all surgeons I have been greatly annoyed by this so-called Colles' fracture, but the fractures differ so much at the lower end of the radius and ulna that if we think of it as a fracture of any bone it is perhaps of the bones of the hand, because it occurs much more frequently than we think, and to depend entirely upon getting the part in the proper position at the time under an anæsthetic I believe is the most we can do for our patients.

DR. SAWYER: We have the operation of the styloid process. Some years ago Dr. Moore stated that he recognised this separation of the styloid, and his treatment, or at least the treatment that he gave his own son, who is now living, was the one that was suggested, that where the disability arose from the impinging of the process, to remove it, and I believe he did remove it in certain cases.

E. W. MULLIGAN, of Rochester: Dr. Moore was very much interested in this subject of the *x*-ray before he died. He was getting to be an old man at that time, and he suffered a cerebral hemorrhage, but his mind was very clear. He asked me a great many times if I had seen the fracture of the end of the ulna, the tip end of the ulna fractured, and I assured him that in a great many cases we did. He found in a majority of cases the tip broken off. Of course Dr. Weigel will tell you in a little while why he thinks we have difficulties with this Colles' fracture that we have been unable to recognise heretofore, due to atrophy of the socket. I have seen this fracture reduced perfectly and watched it with the *x*-ray, and have then later seen results which were not so good. It was undoubtedly due to the condition which Dr. Weigel will speak of later on, bone atrophy. The *x*-ray does not show perfectly the condition of the bone, and if the practitioner depends upon it to show him that, he will oftentimes be mistaken. I would not depend on an *x*-ray to tell me certainly. I would depend on a supplemental examination. Dr. Moore was a great man and he has not been appreciated as he ought to have been. I have noticed that in reading books on surgery his name is omitted in a majority of text-books. I believe now he will be recognised as a great man in the future. His teachings will be shown to be correct. He did not have the *x*-ray, he could not tell all about that, but he knew more about it than any other man in his lifetime. He will be honored as time goes on, without doubt.

LEWIS W. ROSE, of Rochester: The importance of not too much manipulation in the reduction of these fractures is to be further emphasised, I think, because there is often quite a separation in the styloid. There have also been *x*-ray pictures taken where the styloid was separated, and no evident fracture of the radius. Dr. Moore does not receive credit in the modern text-books of surgery and works on practise for his work, and I think Sand's Surgery is the only one at the present day which makes proper note of the work of Dr. Moore on Colles' fracture.

D. N. TOTMAN, of Syracuse: I do not wish to state in my paper that the treatment of Dr. Moore with the splint was at all necessary. I do not know how I could do without the roller under the head of the ulna and the rubber bandage. I do not see how that can be dispensed with in any case, and I want to make that point prominent; if you go on without that adhesive plaster and roller you cannot hold the bones in place. I believe that it is an imoprtant factor in holding the bones in place. Dr. R. W. Moore would take off this roller at the end of two weeks. I keep it on at least four weeks, and sometimes five weeks, leaving the hand free to have the motion, and to have the fingers free. From the very beginning I insist on that motion. In the mater of the removal of this styloid process, I do not wish to say that that loose piece of the bone is much of any factor. Dr. Moore said that when he had a compound fracture he generally removed it, and such operations of the lower extremity of the ulna is sometimes wise, but I never heard of Dr. Moore going into a sound wrist and removing it. It is the injury of the periosteum about the bone that may be the factor. In speaking of the text-books, the very reason that I wrote this paper and brought up this discussion lay in the fact that the text-books used in the medical colleges do not teach true on this subject, and even Dr. Sand's book is not a true book and he has not given Dr. Moore just credit, and I would not use his book as a text-book.

L. A. WEIGEL, of Rochester, read a paper entitled "Bone Atrophy as a Sequel of Traumatism."

DISCUSSION.

A. A. JONES, Buffalo: Does flat foot frequently result from this cause?

THOMAS JAMESON, of Rochester: I think this matter is very important from a medico-legal standpoint. Very often persons are injured in a street car or railroad accident, and we do not get good results under treatment. They develop pain and tenderness, and I think it is well for us all to understand that these people are not all pretending, some of them are injured, although we are not able to detect it at the time.

EDWARD MULLIGAN, of Rochester: I wish to call attention to one point, that in cases of bone atrophy, where we get poor results, the surgeon called later is not justified in thinking that the surgeon first called was at fault. I believe that the majority do not understand, and I wish they could be made to see it more clearly, and I am sure that very many Colless fractures that have bad results are due to this thing and we have never recognised it before.

L. A. WEIGEL, Rochester: I will answer the question about the production of flat foot. I do not believe this bone atrophy is the cause of flat foot.

WILLIS E. FORD, of Utica, read a paper entitled "Some of the Surgical Accidents of Childbirth."

The president: The paper of Dr. Ford's is before us for discussion. It has been suggested by the Business Committee that we have Dr. Mulligan's paper on Cesarean Section read before Dr. Ford's paper is discussed and then have them discussed together.

EDWARD W. MULLIGAN, Rochester, read a paper entitled "Cesarean Section.

A. B. MILLER, of Syracuse: In the paper of Dr. Ford, he had as much in one case as the majority of us get in six, where we have to contend with surgical complications following childbirth. I am inclined to think there are very few men present today who have seen cases of this character. It is an exceptional case. I believe our younger men today, as they get out of college and are prepared to cope with these cases, would recognise it was an unusual case and could take Cesarean Section as a natural route. The injury to the bone certainly is a rare one.

THOMAS JAMESON, Rochester: I would like to relate a little experience of Cesarean Section in the country. I was called out from Rochester one afternoon, and I had just forty minutes to get the car that goes to Williamson. The doctor told me he had a young woman who had been in labor forty-eight hours, and he decided that he had to perform Cesarean Section. I did not have much time to get ready for the operation. I managed to get a nurse, and went down there and found the young woman in a hotel in a very serious condition, her pulse very rapid, and she had lost blood in an attempt at delivery by four different physicians. We got a room ready as soon as possible. I thought it was useless to try to deliver her through the vagina, especially as I could not feel anything through the cervix. Without further delay we got the room ready as rapidly as possible in the hotel and I operated on her and delivered the child. The head and feet of the child were up in the upper end of the uterus and the abdomen was pressed down to the cervix. Her condition was such that I merely delivered the child, which was dead, and sewed up the uterus. She made quite a rapid recovery, although we got some suppuration. That was a year ago. I saw her down at the village the other day and she was in perfect health. She would not let me examine her, but told me she was perfectly well, although I believe from this suppuration there may have been some adhesion to the abdominal wall. This case I think is unique, inasmuch as it was done in a great hurry and under very disadvantageous conditions, although we were as careful as possible.

N. G. Richmond, Fredonia: It seems to me the presentation of these four papers must mark this as a very distinctive meeting, and I am afraid the scene Dr. Ford has portrayed is too often pictured in the country home of today. It is too true that a man at the end of several hours gets in a state of desperation, and perhaps without sufficient preliminary examination to have made a proper diagnosis, and he feels something must be done, and he uses the force which results in such a deplorable condition of affairs. Another thing I regret is the fact that we not only do not have papers of this kind often enough, but that the average practitioner is looking upon this class of work as something another man would better do, and the average practitioner often calls the stranger from a distance to attend the obstetrical cases. I believe obstetrics should have a wider field and we should have more of a deire to do good obstetrical work, and it should be more general in the profession than it is today.

Dr. Loeb: Dr. Ford's paper is certainly very interesting, for it sounds a word of warning to every man to remember that in obstetrical work prevention is worth twenty-five times as much as cure. It is the duty of a medical man at the present time to advise his clientele to consult him promptly. People come to him, not as a usual thing in the beginning, but after seven or eight months have elapsed, and when anything is wrong it is difficult to correct. He should educate the women to come to him as soon as they are pregnant, and it is the duty of the physician to consider that woman his patient until the end. He must be careful to examine her once or twice a month or so before the time of her delivery is expected, so as then to determine whether any dystocia or other trouble exists. You can go on in that line for an hour and talk about the duty of the physician. Another thing I wish to call attention to the physicians who are likely to be called upon to do a Cesarean Section rather rapidly, that when the practitioner gets into the uterus he should first diagnosis where the placenta lies, for I recently saw a case where a child was exsanguinated by the operator going through the placenta, which would not have occurred had he waited for it to be expelled.

Willis E. Ford: There are two points of view from which we look at these injuries. One, of the practitioner who is called hurriedly to a case of labor at some distance and finds himself confronted by a terrible accident, perhaps the result of his want of skill, and perhaps not. He finds himself with a rent as bad as this one I saw. Should he under such circumstances proceed to sew up, should he proceed to a major surgical operation, or should he have skill enough and pluck enough to use iodoform gauze until the surgeon comes? Certainly where you find a tear in the uterus he has no business to do it on the spot. It seems to me to open and take out the uterus is not only a mistake but a crime. The other point is where a patient is in a lying-in hospital and is in the hands of a skilled obstetrician all the time. Of course he knows the measurements of the pelvis. He can select his operation. That situation I do not deal with at all. I am simply talking to the general practitioner. It seems to me the skillful packing of the gauze is a much safer operation than to proceed with a set operation.

E. W. Mulligan, Rochester: Cesarean Section should be done more frequently than it is. We have a great many children destroyed and a great many mothers killed because of the bad practice of the attending obstetrician. It is more difficult to deliver a woman with forceps than it is to do an abdominal section, and yet every man is doing it. I do not think I ever tore a sphincter clear through in my life delivering a woman—yes, I did once—but I can understand how that thing might happen, and any man who says such a thing should never happen is a man who has not had much experience.

Lucien Howe, of Buffalo, gave a demonstration of the effects of dionin on the eye, presenting two cases.

Floyd S. Crego, of Buffalo, read a paper entitled "Lumbar Puncture and Lymphocytosis with Report of Work done in Germany by Author during Past Year."

Allen A. Jones, Buffalo, read a paper entitled "Treatment of Chronic Catarrhal Gastritis."

A. A. Young, Newark, read a paper entitled "Infantile Indigestion."

DISCUSSION

N. G. Richmond, Fredonia: I have been particularly interested in these papers, and I would like to add emphasis to some things that have been stated, and refer to some things which have not been, stated particularly in the last paper. Reference was made to bacteria. I believe that they are not found in the gastro-intestinal tract at birth, but after 24 hours. There are two varieties found in the intestinal tract. Their function is not known. The bacteria are not known. The bacteria present a very important point in the food taken by the infant. It seems to me more stress might be laid upon the precautions to be taken with cow's milk, and that is in reference to the cleanliness, and subjecting it to cooling influences. Twenty-three *kinds* (?) of bacteria are said to be found in milk improperly cooled, and these bacteria are the ones which result in the early interference with digestion. It seems to me that much milk would be acceptable which we condemn if it could be obtained with the cleanliness which we know is essential and if the milk be properly cooled. Inasmuch as the stomach of the infant does not assume the horizontal position immediately and is something like a pail from which the water is spilled easily, is seems to me this is nature's method of relieving the stomach of unwelcome material. I would like to emphasise the summing up of the doctor in regard to the withholding of food and increasing the water. It seems to me that many times the infant requires water, and the mother invariably answers it by food.

The president: The next paper is "Tumors of the Neck", by Dr. Rose of Rochester.

L. W. ROSE, Rochester: With your permission I will change the title from "Tumors of the Neck" to "Tumor of the Neck," and present a photograph which recently came under my observation.

D. H. MURRAY, Syracuse, read a paper entitled "Some Minor Surgical Questions in Rectal Surgery."

The president: I have to announce that the morning is about to close and we are about to take a recess. We are to re-assemble at a quarter before three on the eastern exposure, where a photograph of the assembly is to be taken. That function is to occur at 2:45. We will go to lunch at the Park Club directly across the road from the Historical Building where I think most of you have been before. The morning session now stands adjourned.

